# Changes in cardiorespiratory fitness and activity levels over the first year after discharge in ambulatory persons with recent incomplete spinal cord injury

**DOI:** 10.1038/s41393-020-0514-7

**Published:** 2020-07-09

**Authors:** Matthijs F. Wouda, Eivind Lundgaard, Frank Becker, Vegard Strøm

**Affiliations:** 1 Department of Research, Sunnaas Rehabilitation Hospital, Oslo, Norway; 2 University of Oslo, Institute of Clinical Medicine, Oslo, Norway

**Keywords:** Neurophysiology, Spinal cord diseases

## Abstract

**Study design:**

Secondary analysis of a clinical trial.

**Objectives:**

To investigate changes in cardiorespiratory fitness (CRF) and activity level in ambulatory persons with SCI during the first year after discharge from inpatient rehabilitation.

**Setting:**

Sunnaas Rehabilitation Hospital, Nesoddtangen, Norway.

**Methods:**

Thirty persons with incomplete SCI, all community walkers (25 males and 5 females, 18–69 years old) were recruited to a clinical trial of a 12 weeks home-based aerobic exercise program of either high or moderate intensity. During the last week of inpatient rehabilitation (baseline), participants performed a maximal exercise test on a treadmill (peak oxygen uptake; peak VO_2_) and a 6-min walking test (6MWT). Also, total daily energy expenditure (TDEE) and daily amount of steps were measured continuously during 7 days in the participants’ homes. All tests were repeated after 3 and 12 months (post tests).

**Results:**

Twenty of the 30 clinical trial participants performed baseline and both posttests and are included in this secondary analysis. We found no statistically significant between-group differences in the time course over the first year of either peak VO_2_, 6MWT, or physical activity outcomes. Therefore, data from both exercise groups and the control group were merged for secondary analyses, revealing statistically significant increase over time in peak VO_2_, 6MWT, and TDEE. The increase over time in the average daily steps did not reach statistical significance.

**Conclusions:**

Ambulatory persons with SCI were able to increase their CRF levels over the first year after discharge from inpatient rehabilitation, despite a minimal increase in activity levels.

## Introduction

Persons with spinal cord injury (SCI) often have low levels of daily physical activity [1, 2] and reduced cardiorespiratory fitness (CRF) [3–5]. Physical activity can be defined as any bodily movement produced by skeletal muscles that require energy expenditure [6]. CRF reflects the integrated ability to transport oxygen from the atmosphere to the mitochondria to perform physical work. CRF quantifies an individual’s functional capacity and can be measured directly, expressed as maximal oxygen consumption (VO_2_ max) or peak oxygen consumption (peak VO_2_) [7].

Being physically active seems to increase CRF levels and improve health factors in persons with SCI [8]. After discharge from inpatient rehabilitation, physical activity levels, however, seem to decrease in persons with a complete SCI [9]. This might lead to physical deconditioning that can further exacerbate the impact of the injury and lead to an increased risk of cardiovascular disease [10].

In Norway, the specialized SCI rehabilitation units have a commitment to life-long follow-up for persons with SCI. After discharge from the first rehabilitation period, they are admitted for regular medical checkups (3–5 days inpatient care) depending on their needs. In addition, they can contact the hospital if they have specific issues that need solving [11]. In 2018, 125 persons with a newly acquired SCI were registered in Norway, of which 48 (38%) had a preserved/regained walking function without the need for walking aids when they were discharged from inpatient rehabilitation [12]. Similar to those who are wheelchair dependent, ambulatory persons with incomplete SCI seem to be inactive [13] and show lower levels of physical fitness [4, 14] compared to able-bodied (AB) persons. Those with an incomplete SCI have potentially more intact muscle groups below the injury level that can be utilized during physical activity. Therefore, persons with incomplete SCI, especially those with partially preserved or regained walking function, might have better premises to achieve high levels of physical fitness.

Previously [15], we reported that 30 ambulant persons with SCI increased their CRF, but not their physical activity levels during the first 3 months after discharge from inpatient rehabilitation. It is uncertain, however, if participants are able to maintain CRF levels and physical activity levels. Therefore, the purpose of the present study is to describe the changes in CRF and activity levels during the first year after inpatient rehabilitation in a group of ambulatory persons with incomplete SCI.

## Methods

### Design

Participants were originally recruited to take part in a 12-week RCT, including a follow-up after 1 year [15]. They performed a home-based aerobic exercise intervention that consisted of either high-intensity interval training (HIIT), moderate-intensity training (MIT), or “treatment as usual” (control group). The MIT group was instructed to walk/run three times a week, for 45 min continuously at 70% of their peak heart rate (peak HR), while the HIIT group had to walk/run at an intensity of 85–95% of peak HR interspersed with 3 × 3 min recovery periods at an intensity of 70% of peak HR. The training mode was either walking or running (on a treadmill or outdoors) depending on the participants’ CRF level and physical constraints. Due to ethical considerations, participants in the control group were not prescribed any aerobic exercise, but had no restrictions regarding physical training. All participants could if needed have contact with health care providers in the community (e.g., a physiotherapist). A detailed description of the recruitment, the interventions, and the dropout rate during the first 3 months after discharge has been provided in a previous paper [15].

### Participants

In Norway, the average length of hospital stay from acute care until the end of inpatient rehabilitation was 116 days for traumatic SCI in 2018 [12]. Thirty participants (25 men and 5 women) were recruited during the last two weeks of inpatient rehabilitation by consecutive enrollment over a 4-year period (2013–2017). Inclusion criteria were persons between 18 and 17 years of age, with traumatic or nontraumatic SCI of all lesion levels [16] that had regained the ability to walk independently in the community (“community walkers”), defined by being able to walk on a treadmill for 5 min at 3 km/h (without assistive walking aids). In addition, they had to be in their final phase of the subacute inpatient rehabilitation program at Sunnaas Rehabilitation Hospital. There were no restrictions for inclusion with regard to wheelchair use. Participants were excluded if they had significant concurrent medical conditions that might limit their CRF (e.g., psychiatric conditions, orthopedic diseases, or uncontrolled cardiopulmonary disease). Although the intended sample size (*n* = 45) was not accomplished, recruitment had to be ended due to time constraints. Due to lack of motivation (*n* = 6), pain (*n* = 3), and comorbidity (*n* = 1), 10 participants were lost to follow-up after 12 months. Thus, only 20 out of 30 participants performed all tests at baseline and at 3 and 12 months after discharge from inpatient rehabilitation.

### Procedures

After medical approval for inclusion, (baseline) CRF tests were performed during the last week of the inpatient rehabilitation, comprising a maximal treadmill exercise test and a 6-min walking test (6MWT). The first week after discharge, participants’ physical activity levels were monitored by wearing a portable activity monitor. This activity monitor, mounted on the right upper arm, was worn for seven consecutive days in a private home situation. Then, the 12-week intervention period started the second week after discharge. Three months (i.e., after the intervention period) and 1 year after discharge from inpatient rehabilitation, participants returned to the hospital for the CRF tests, repeating the same procedures as for the baseline tests. Physical activity was monitored during the 7 days after they had returned to the hospital.

### Outcome measures

CRF was determined by measuring peak VO_2_ (prespecified primary outcome measure) using a computerized standard open-circuit technique breath-by-breath spirometer (Vmax 220, Sensormedics Corporation, Yorba Linda, CA, USA) during a maximal graded exercise test on a treadmill (Woodway PPS Med, Waukesha, WI, USA). In addition, respiratory exchange ratio (RER), peak HR (Polar M400), and blood lactate [La-] (BIOSEN C-line, Sport, EFK diagnostics, Barleben, Germany) were measured to evaluate whether criteria for maximal exercise testing were achieved; RER (>1.15), peak HR > 85% of expected (for men: >220–0.88 × age, for females: 220–0.66 × age) and [La-] (>8.0 mmol/L) [17]. A modified Sunnaas protocol for maximal exercise testing on a treadmill was used [4]. After establishing a self-selected walking speed, the inclination of the treadmill was increased every minute by 2%, up to 20% inclination or until exhaustion. If exhaustion was not reached at the self-selected speed and 20% inclination, the speed was increased every minute by 0.5 km/h until exhaustion. VO_2_ was time-averaged over 30 s. The highest time-averaged value was considered peak VO_2_.

Participants performed the 6MWT (secondary outcome measure) to evaluate their walking capacity. We registered the number of meters walked, and the heart rate after the test (HR_after-test_), measured with a heart rate monitor (Polar M400, Kempele, Finland).

Physical activity levels were measured with the SenseWear™ Pro_2_ Armband (SWA) (Bodymedia Inc., Pittsburgh, PA, USA), a small activity monitor attached on the right upper arm. The total daily energy expenditure (TDEE) (kilojoule (kJ) per minute) and the daily number of steps were measured. TDEE results were divided by 41,858 to transform TDEE into kilocalories (kcal) per minute [18]. SenseWear Professional 7.0 (software) was used to analyze the activity monitor data.

### Data analyses

Statistical analyses were performed with the Statistical Package for the Social Sciences (release 25.0.0.1 SPSS Inc., Chicago, IL, USA). Descriptive results for all outcome measures at three different time points (baseline, 3 months post discharge and 12 months post discharge) are presented as means and standard deviations (SD).

We used linear mixed model analysis for repeated measurements, to fit the best model for the time course (i.e., modeling the response of the dependent variables peak VO_2_, 6MWT, TDEE, and daily steps, respectively). All 30 participants recruited in this study were used in this analysis. In this model, time was treated as a repeated fixed factor, allowing for individual random intercept and slope, and fitted with a variance components covariance structure. A time^2^ term was added to the models to test for a possible nonlinear response. Model selection was based on the Akaike information criterion (AIC) (i.e., a lower AIC represents a stronger model).

## Results

Baseline demographics and injury-specific characteristics of all participants (*n* = 30) that were originally enrolled in the study [15] and of the remaining 20 participants at 12 months follow-up are described in Table 1. The characteristics of those lost to follow-up at 12 months (*n* = 10) did not seem to differ substantially from those that fulfilled all tests. None of the participants in this study reported that they had used a wheelchair during the first year after discharge.

**Table 1:** Partcipants demographics and injury-specific characteristics at baseline and after 12 months.

	Baseline (*n* = 30)	12 months (*n* = 20)
Demographics		
Age (years; mean (SD))	41 (17)	42 (17)
Body weight (kg; mean (SD))	81 (16)	82 (18)
BMI (kg/m^2^; mean (SD))	25.4 (4.2)	26.0 (4.6)
Smoking (*n*)	5	2
Male:Female (ratio)	25:5	16:4
Injury-specific characteristics		
Traumatic:nontraumatic (ratio)	24:6	16:4
Time since injury (days; mean (SD))	69 (29)	–
*Neurological level*^*a*^ (*n*)		
Cervical 1–8	18	12
Thoracic 1–5	3	3
Thoracic 6–12	3	1
Lumbar 1–5	5	4
Sacral 1–5	1^b^	–

*SD* standard deviation, *BMI* body mass index.

^a^Neurological injury level (International Standards for Neurological Classification of Spinal Cord Injury).

^b^Apart from this participant (ASIA-A, SCI at level S2), all participants had an incomplete SCI, ASIA-D.

Descriptive results from the physical exercise tests and the physical activity monitoring for each group at the different time points are presented in Supplementary Table 1. No statistically significant differences were found between the groups in the time course of either peak VO_2_, 6MWT, or physical activity outcomes (see Supplementary Table 2). Therefore, data from the three groups were combined in the following analyses.

Participants’ mean peak VO_2_ (in both l/min and ml/kg/min) was higher, and the distance covered during the 6MWT was longer, at both 3 months and 12 months post discharge compared to baseline (Table 2). The activity monitoring data (i.e., TDEE and daily number of steps) revealed similar results (Table 2).

**Table 2:** The mean results (with SD) from the maximal exercise test, 6MWT, and physical activity monitoring at baseline, 3 and 12 months.

Physical capacity	Baseline Mean (SD)	3 months Mean (SD)	12 months Mean (SD)
Graded maximal exercise test	(*n* = 29)	(*n* = 25)	(*n* = 19)
Peak VO_2_ (l/m)	2.76 (0.71)	3.13 (0.72)	3.03 (0.67)
Peak VO_2_ (ml/kg/m)	34.9 (9.2)	39.5 (8.8)	38.0 (9.4)^a^
Peak HR (beats/m)	175 (18)	180 (15)	179 (15)
RER (ratio)	1.21 (0.11)	1.21 (0.07)	1.18 (0.06)
[La-] (mmol/l)	8.9 (2.8)	9.0 (2.8)	7.8 (2.1)
Maximal speed (km/h)	5.0 (1.0)	5.6 (1.4)	5.8 (2.1)
Maximal angle (%)	15.7 (4.4)	17.0 (3.2)	15.8 (4.7)
6MWT	(*n* = 30)	(*n* = 25)	(*n* = 20)
Distance (m)	581 (89)	658 (106)	672 (91)
HR_after-test_ (beats/m)	135 (25)	142 (24)	148 (23)
Physical activity			
Activity monitoring	(*n* = 24)	(*n* = 22)	(*n* = 17)
TDEE (kcal)	2632 (509)	2739 (412)	2750 (443)
Daily steps	5724 (2786)	5872 (2330)	6431 (2891)

*SD* standard deviation, VO_2_ oxygen uptake, *HR* heart rate, *RER* respiratory exchange ratio, *La*- blood lactate, *6MWT* 6-min walking test, *TDEE* total daily energy expenditure, *kcal* kilocalories.

^a^At 12 months post discharge, the participants’ mean body weight had increased by 2.6 kg.

**Fig. 1:**
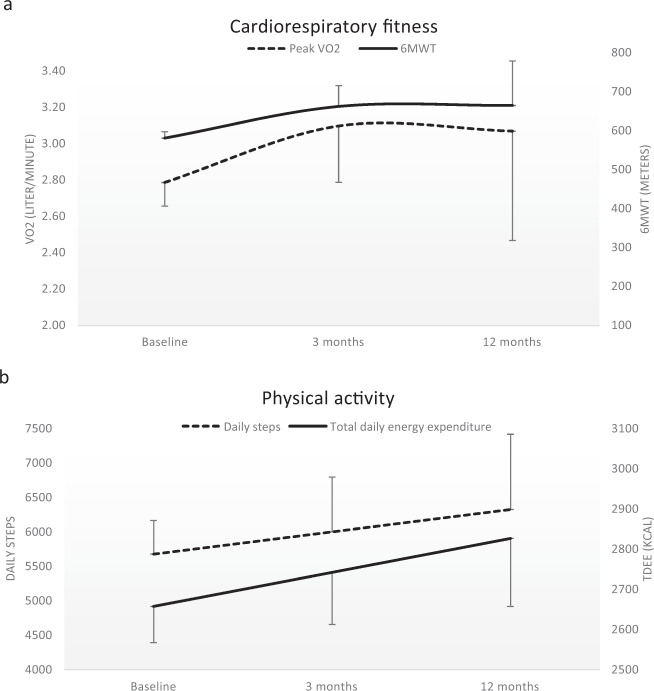
Time course of cardiorespiratory fitness and physical activity parameters. Mixed model parameter estimates (±standard errors of the mean) of fixed effects in the best-fitted models of the time course for **a** peak oxygen uptake (VO_2_, l/min) and 6-min walking test (6MWT, m), and **b** daily number of steps and total daily energy expenditure (TDEE, kilocalories; kcal), over the first year after discharge from inpatient rehabilitation in a group of ambulatory persons with spinal cord injury.

The mixed model analyses of peak VO_2_ (l/min and ml/kg/min) and 6MWT (meters) revealed statistically significant linear increases over time (Table 3). However, significant effects of time^2^ for these outcomes signifies a nonconstant rate of changes across time (Table 3). Plotting the estimated regression coefficients (i.e., the best-fitted models of the time course) of peak VO_2_ (liter/min) and 6MWT (meters) indicate a slight decline/leveling off after peaking at 3 months (Fig. 1a).

**Table 3:** Time course of peak VO_2_, 6MWT, and physical activity outcomes in the participants (*n* = 30).

	Estimates	SE	95% CI
Peak VO_2_ (l/min)			
(*ß*_1_) Intercept	2.79***	0.13	2.52–3.06
(*ß*_2_) Time	0.48**	0.12	0.23–0.73
(*ß*_3_) Time^2^	−0.17**	0.06	−0.29 to −0.05
Peak VO_2_ (ml/kg/m)			
(*ß*_1_) Intercept	35.1***	1.6	31.7–38.4
(*ß*_2_) Time	5.5**	1.5	2.4–8.7
(*ß*_3_) Time^2^	−2.1**	0.7	−3.7 to −0.6
6MWT (m)			
(ß_1_) Intercept	581***	16	547–614
(*ß*_2_) Time	120***	25	72–169
(*ß*_3_) Time^2^	−39**	12	−63 to −16
TDEE (kcal)			
(*ß*_1_) Intercept	2657***	90	2472–2841
(*ß*_2_) Time	84*	39	2–166
Daily steps (number)			
(*ß*_1_) Intercept	5670***	488	4674–6667
(*ß*_2_) Time	323	302	−298 to 943

Estimated regression coefficients (*ß*), standard errors (SE), and 95% confidence intervals (CI) of the linear mixed effect models for repeated measurements fitted with variance components covariance structure of the time course over the first year after discharge from inpatient rehabilitation.

*ß*_1_ = estimates of baseline values; *ß*_2_ = estimates of the change per time unit; *ß*_3_ = estimates of the quadratic change per time unit. Time was in the model categorized as 0: Baseline; 1:3 months post discharge; and 2:12 months follow-up. As an example, the best-fitted model of the time course of peak VO_2_ = *ß*_1_ + *ß*_2_Time + *ß*_3_Time^2^.

*SE* standard error, *CI* confidence interval, VO_2_ oxygen uptake, 6*MWT* 6-min walking test, *TDEE* total daily energy expenditure, *kcal* kilocalories.

*Statistically significant at a level of *p < 0.05; **p < 0.01; ***p < 0.001.

For the physical activity data, the time course analyses revealed a statistically significant linear increase over time for TDEE (kcal), but not for daily number of steps (Table 3). Figure 1b depicts the estimated regression coefficients (see Table 3), suggesting a minor linear increase in both TDEE and daily steps.

## Discussion

The present study is a follow-up to an RCT that included an exercise intervention. The main findings were that over the first year after discharge from inpatient rehabilitation, the participants significantly increased their peak VO_2_ and the distance walked during the 6MWT. The increase over time in the average daily steps did not reach statistical significance, despite a minor, but significant increase in the TDEE.

### CRF and walking capacity

To our knowledge, no other studies have described the development of CRF in ambulatory persons with SCI over the first year after discharge from inpatient rehabilitation. The time course of the peak VO_2_ measures, with the most profound increase after the first 3 months (Fig. 1a), was as expected. The participants had been exercising extensively during the first 3 months after discharge from the hospital, due to enrollment in an RCT of home-based aerobic exercise interventions [15]. Most of the participants that originally were enrolled in the control group reported that they also had been exercising considerably during the intervention period [15]. This might explain why participants in all groups increased their CRF during the first 3 months [15]. The levelling off in peak VO_2_ after 3 months indicates that the participants most likely continued regularly exercising, possibly at moderate or high intensity, to maintain these CRF levels also at 12 months follow-up. Unfortunately, we do not have information about which exercise was performed from 3 months until 12 months post discharge. The increased peak VO_2_ was concomitant to increased walking distances at the 6MWT. The observed increase of 91 m on the 6MWT 1 year after discharge can be considered a clinically meaningful improvement of the walking capacity of the participants [19]. However, the HR_after-test_ was considerably higher at 12 months follow-up, compared to baseline values (Table 2). Thus, the participants were able to walk at higher aerobic intensity one year after discharge, possibly due to the increased CRF levels. Increased lower extremity muscle strength and motor control and less spasticity might have also been contributing factors; however, we did not measure these.

The participants in the present study exhibited relatively high levels of physical fitness, as they had only 10% (4.6 ml/kg/min) and 5% (2.6 ml/kg/m) lower peak VO_2_ levels at baseline and at 1-year follow-up, respectively, compared to (Norwegian) AB persons [20]. Furthermore, the mean distance walked during the 6MWT was, when compared with AB persons (i.e., age 20–59, males: 748 m, females: 693 m) [21], slightly reduced at baseline and at a similar level at 1-year follow-up. Most of the participants in the current study reported regularly exercising the three months prior to their SCI [15]. It is plausible that participants with high physical fitness, high interest in physical activity, and established training habits before the injury were more likely to participate in our study.

A few cross-sectional studies [4, 13, 14] have measured fitness levels in ambulatory persons with incomplete SCI. Two studies [4, 14] on ambulatory persons with SCI, in which comparable inclusion criteria and test protocol were used, showed similar CRF levels compared to the present study.

Saraf et al. [13] included 25 persons with incomplete SCI, classified as “community walkers.” Besides a significantly longer time post-injury (mean: 79 months), the injury-specific characteristics and demographics of the participants in the study of Saraf et al. are comparable to the present study. Participants in their study were, however, less physically fit and performed poorer during the 6MWT compared to the baseline results of participants in the present study. The discrepancies may partly be explained by the different inclusion criteria that were used. One of the criteria in the present study, being able to walk 5 min at 3 km/h without walking aids, might demand a higher function of the participants, than those included in the study of Saraf et al. [13].

### Physical activity

The significant time effect of TDEE could be due to the participants’ gain in body weight, combined with a small increase in daily steps. However, due to the rather low accuracy of the SWA, especially when estimating energy expenditure during exercise [22], this small but significant change in TDEE cannot automatically be considered as clinically meaningful. The lack of significant time effect in the number of daily steps found in our study might be due to large between-subject variance (Table 2). As we found significantly increased CRF levels over the time course, a similar increase in daily steps could be expected. However, as demonstrated by Zisko et al. [23], in AB persons, higher CRF levels do not necessarily lead to more daily steps or an increase in TDEE. Even though the increased values did not reach statistical significance, it might be of clinical importance. The increase of about 700 steps/day from baseline to 12 months follow-up is, however, lower than a lower-bound estimate of a clinically meaningful change of ~800 steps/day in free-living walking behavior in persons with Multiple Sclerosis [24]. Barriers (e.g., lack of training facility) and facilitators (e.g., social contacts) might have influenced the participants’ physical activity levels [25]. Some researchers have proposed that a reduced lower extremity muscle strength limits the physical activity level in the ambulatory SCI population [26, 27]. Participants in our study might have focused mainly on the (prescribed) aerobic exercise and thereby neglected muscle strength exercise. Ginis et al. claimed that ambulatory persons with SCI show poorer attitudes toward leisure-time physical activity than those who are wheelchair dependent [28]. As to our knowledge, only one other study has monitored physical activity levels of ambulatory persons with SCI over the first year after discharge from inpatient rehabilitation [29]. They found that participants’ daily physical activity levels changed favorably during the first half-year after inpatient rehabilitation and remained stable during the second half-year, thus not unlike our time course results.

The average level of ~6000 steps per day found in our participants are somewhat below the 7100 steps/day (performed at moderate intensity) that is recommended in older adults and individuals with a disability or chronic disease [30]. They, however, emphasize that individuals living with disability and chronic illness may be more limited in their everyday activities, but could still benefit from a physically active lifestyle approximating 4600 steps/day if averaged over a week of free-living behavior [30].

### Limitations

Some limitations warrant comments; first, the low sample size of 30 participants (20 at 12 months follow-up), was smaller than the intended sample size of 45, resulting in low statistical power. The results must therefore be interpreted with caution. Second, generalizing our results should be limited to the community walking SCI population, i.e., those who are able to walk without walking aids. Third, the activity monitor (SWA) used in our study has not been validated in persons with incomplete SCI, thus caution is warranted when comparing the results on TDEE and daily steps with other populations. Fourthly, the SWA data on physical activity was collected only during seven days and thus might not reflect the participants’ habitual activity levels.

To conclude, a group of ambulatory persons with SCI exhibited increased CRF levels and walking capacity over the first year after inpatient rehabilitation. This increase was most evident after the first three months, subsequent to participation in a home-based physical exercise intervention. The findings suggest that ambulatory persons with SCI may have the potential to increase and then maintain their physical fitness levels after being discharged. Physical activity levels remained rather low during the first year post discharge. This might indicate that there is a need for promoting physical activity in persons with ambulatory SCI after being discharged from inpatient rehabilitation.

## Supplementary information

Supplementary table 1 and 2

Dataset 1

## Data Availability

All data generated or analyzed during this study are included in this published article (and its supplementary information files).
