# Supplementary material for: Changes in cardiorespiratory fitness and activity levels over the first year after discharge in ambulatory persons with recent incomplete spinal cord injury
Source: Spinal Cord. 2020 Jul 9;59(3):354–60. doi: 10.1038/s41393-020-0514-7 (PMC7943418; doi:10.1038/s41393-020-0514-7)
Supplement: Supplementary file 1 — Supplementary table 1 and 2 [file 41393_2020_514_MOESM1_ESM.pdf]

Supplementary table 1. Results on the maximal exercise test, 6MWT and activity monitoring for all participants that were recruited to the randomized controlled trial (n=30). The table shows descriptive data for each group (HIIT, MIT and control group) at the three time points (at baseline, at three months post-discharge and at one-year follow-up).

	**HIIT**			**MIT**			**Control**		
	**Baseline**	**3 months**	**12 months**	**Baseline**	**3 months**	**12 months**	**Baseline**	**3 months**	**12 months**
**Graded maximal exercise test**	*(n=10)*	*(n=9)*	*(n=8)*	*(n=10)*	*(n=9)*	*(n=6)*	*(n=9)*	*(n=7)*	*(n=6)*
Peak VO_2 (L/min)	2.70 (0.81)	3.00 (0.62)	3.11 (0.74)	2.79 (0.79)	3.23 (0.94)	3.08 (0.71)	2.78 (0.57)	3.15 (0.59)	3.02 (0.69)
Peak VO_2 (ml/kg/min)	32.1 (9.1)	35.7 (5.3)	36.7 (7.7)	36.9 (11.8)	42.3 (12)	40.6 (12.8)	35.6 (6.0)	40.7 (6.5)	37.8 (7.7)
Peak HR (beats/min)	167 (13)	173 (15)	172 (18)	177 (24)	184 (15)	189 (12)	182 (11)	183 (14)	178 (11)
RER (ratio)	1.23 (0.10)	1.21 (0.71)	1.21 (0.02)	1.17 (0.14)	1.19 (0.07)	1.15 (0.08)	1.24 (0.06)	1.22 (0.06)	1.18 (0.06)
[La-] (mmol/liter)	8.96 (2.25)	8.7 (1.2)	7.40 (2.0)	8.99 (3.72)	10.4 (2.8)	9.34 (2.14)	8.78 (2.46)	8.02 (2.44)	7.67 (2.67)
**6MWT**	*(n=10)*	*(=9)*	*(n=8)*	*(n=9)*	*(n=9)*	*(n=6)*	*(n=10)*	*(n=7)*	*(n=6)*
Distance (meters)	561 (93)	654 (96)	674 (88)	573 (96)	678 (136)	714 (75)	607 (77)	638 (80)	626 (101)
$HR_{after\text{-}test}$ (beats/min)	118 (17)	134 (21)	138 (18)	145 (29)	152 (32)	166 (17)	143 (21)	142 (24)	138 (24.6)
**Activity monitoring**	*(n=10)*	*(n=9)*	*(n=8)*	*(n=8)*	*(n=7)*	*(n=6)*	*(n=10)*	*(n=6)*	*(n=5)*
TDEE (Kcal)	2666 (528)	2744 (359)	2908 (440)	2736 (603)	2768 (531)	2706 (523)	2437 (341)	2697 (405)	2558 (276)
Daily steps	5223 (2737)	6217 (3080)	7075 (3568)	6538 (3580)	6125 (2175)	5711 (2708)	5477 (1624)	5057 (922)	6025 (1851)

Abbreviations: Standard Deviation (SD), High-Intensity Interval Training (HIIT), Moderate-Intensity Training (MIT), Oxygen Uptake (VO_2), Heart Rate (HR), Respiratory Exchange Ratio (RER), blood lactate [La-], 6 Minute Walking Test (6MWT), Total Daily Energy Expenditure (TDEE), Kilocalories (Kcal).

Supplementary Table 2. *Estimated regression coefficients, standard errors and 95 % confidence intervals of the linear mixed effect models for repeated measurements fitted with variance components covariance structure of the time course over the first year post-discharge for participants in the high intensity interval training (HIIT), moderate intensity training (MIT) and control groups.*

		Estimates	SE	95% CI	
				Lower	Upper
**Peak VO₂** (l/min)	[a](ß₁) Intercept	2,91***	0,24	2,44	3,42
	[b](ß₂) Group HIIT	-0,21	0,34	-0,90	0,49
	[c](ß₃) Group MIT	-0,08	0,34	-0,78	0,61
	[d](ß₄) Time	0,07	0,11	-0,15	0,29
	[e](ß₅) Time * Group HIT	0,18	0,14	-0,11	0,47
	[f](ß₆) Time * Group MIT	0,04	0,14	-0,26	0,34
**Peak VO₂** (ml/kg/min)	[a](ß₁) Intercept	37,0***	2,9	31,0	43,0
	[b](ß₂) Group HIIT	-4,6	4,1	-13,0	3,9
	[c](ß₃) Group MIT	0,7	4,1	-7,7	9,2
	[d](ß₄) Time	0,2	1,3	-2,6	2,9
	[e](ß₅) Time * Group HIT	2,4	1,7	-1,2	6,1
	[f](ß₆) Time * Group MIT	0,8	1,8	-2,9	4,6
**6MWT** (meters)	[a](ß₁) Intercept	613***	29	553	673
	[b](ß₂) Group HIIT	-41	42	-125	43
	[c](ß₃) Group MIT	-27	42	-112	57
	[d](ß₄) Time	19	16	-14	52
	[e](ß₅) Time * Group HIT	40	21	-4	84
	[f](ß₆) Time * Group MIT	36	22	-10	82
**TDEE** (kcal)	[a](ß₁) Intercept	2558***	173	2204	2912
	[b](ß₂) Group HIIT	110	227	-355	576
	[c](ß₃) Group MIT	187	239	-303	677
	[d](ß₄) Time	65	77	-96	225
	[e](ß₅) Time * Group HIT	85	96	-116	285
	[f](ß₆) Time * Group MIT	-59	103	-275	157
**Daily steps** (number)	[a](ß₁) Intercept	5266***	951	3329	7201
	[b](ß₂) Group HIIT	-35	1230	-2547	2476
	[c](ß₃) Group MIT	1349	1295	-1290	3988
	[d](ß₄) Time	292	582	-906	1489
	[e](ß₅) Time * Group HIT	568	730	-940	2077
	[f](ß₆) Time * Group MIT	-703	788	-2329	924

Abbreviations: *Standard error (SE), Confidence interval (CI), Oxygen Uptake (VO_2), 6 Minute Walking Test (6MWT), Total Daily Energy Expenditure (TDEE), Kilocalories (kcal).*

[a] estimate of baseline value for the control group; [b] estimate of the difference between HIIT and control group at baseline; [c] estimate of the difference between MIT and control group at baseline; [d] estimate of change per time unit; [e] estimate of difference per time unit between HIIT group and control group; [f] estimate of difference per time unit between MIT and control group.

** Statistically significant at a level of $p<0.05$, ** $p<0.01$, and *** $p<0.001$*
